# Comparison of methods to detect the in vitro activity of silver nanoparticles (AgNP) against multidrug resistant bacteria

**DOI:** 10.1186/s12951-015-0120-6

**Published:** 2015-10-05

**Authors:** Emerson Danguy Cavassin, Luiz Francisco Poli de Figueiredo, José Pinhata Otoch, Marcelo Martins Seckler, Roberto Angelo de Oliveira, Fabiane Fantinelli Franco, Valeria Spolon Marangoni, Valtencir Zucolotto, Anna Sara Shafferman Levin, Silvia Figueiredo Costa

**Affiliations:** Department of Infectious Diseases, University of São Paulo, São Paulo, Brazil; Department of Surgery São Paulo, University of São Paulo, São Paulo, Brazil; Faculty of Engenier São Paulo, University of São Paulo, São Paulo, Brazil; Nanomedicine and Nanotoxicology Group, University of São Paulo, IFSC, São Paulo, Brazil; LIM-54 (Laboratório de Bacteriologia), Instituto de Medicina Tropical, Av. Dr. Enéas de Carvalho Aguiar, 470, Prédio II, 1º andar, Sala 112, Cerqueira César, São Paulo, SP 054503-00 Brazil

**Keywords:** Silver nanoparticle, Antimicrobial test, Multidrug resistant bacteria

## Abstract

**Background:**

Multidrug resistant microorganisms are a growing challenge and new substances that can be useful to treat infections due to these microorganisms are needed. Silver nanoparticle may be a future option for treatment of these infections, however, the methods described in vitro to evaluate the inhibitory effect are controversial.

**Results:**

This study evaluated the in vitro activity of silver nanoparticles against 36 susceptible and 54 multidrug resistant Gram-positive and Gram-negative bacteria from clinical sources. The multidrug resistant bacteria were oxacilin-resistant *Staphylococcus aureus*, vancomycin-resistant *Enterococcus* spp., carbapenem- and polymyxin B-resistant *A. baumannii*, carbapenem-resistant *P. aeruginosa* and carbapenem-resistant Enterobacteriaceae. We analyzed silver nanoparticles stabilized with citrate, chitosan and polyvinyl alcohol and commercial silver nanoparticle. Silver sulfadiazine and silver nitrate were used as control. Different methods were used: agar diffusion, minimum inhibitory concentration, minimum bactericidal concentration and time-kill. The activity of AgNPs using diffusion in solid media and the MIC methods showed similar effect against MDR and antimicrobial-susceptible isolates, with a higher effect against Gram-negative isolates. The better results were achieved with citrate and chitosan silver nanoparticle, both with MIC_90_ of 6.75 μg mL^−1^, which can be due the lower stability of these particles and, consequently, release of Ag^+^ ions as revealed by X-ray diffraction (XRD). The bactericidal effect was higher against antimicrobial-susceptible bacteria.

**Conclusion:**

It seems that agar diffusion method can be used as screening test, minimum inhibitory concentration/minimum bactericidal concentration and time kill showed to be useful methods. The activity of commercial silver nanoparticle and silver controls did not exceed the activity of the citrate and chitosan silver nanoparticles. The in vitro inhibitory effect was stronger against Gram-negative than Gram-positive, and similar against multidrug resistant and susceptible bacteria, with best result achieved using citrate and chitosan silver nanoparticles. The bactericidal effect of silver nanoparticle may, in the future, be translated into important therapeutic and clinical options, especially considering the shortage of new antimicrobials against the emerging antimicrobial resistant microorganisms, in particular against Gram-negative bacteria.

## Background

In the last decade, the emergence of multidrug-resistant bacteria (MDR) became a challenge around the world [1]. Antimicrobial resistance is recognized as a medical problem that increases mortality, morbidity rates, length of stay and cost and has nowadays few therapeutic possibilities [2]. In this scenario, new products such as nanotechnology may have a role to treat infections due to MDR [3].

Nanotechnology is opening possibilities, allowing new solutions with old resources. Nanoscale materials such as silver nanoparticles (AgNPs) have emerged as novel agents because their high surfaces area to volume ratio and the unique chemical and physical properties [4]. Silver nanoparticles have greater efficiency in mediating their antimicrobial activity when compared with silver salts [5, 6]. Because AgNPs acts synergistically in distinct targets it is expected that there will be no interference with antimicrobial resistance mechanisms. Thus, AgNPs has a potential use to treat MDR.

However, the mechanisms by which AgNPs act against bacteria are not yet fully elucidated. It is believed that in aqueous solution biologically active Ag^+^ ions are delivered and promotes the antimicrobial effect [5–7]. Silver nanoparticles interact with three vital components of the cells: (a) peptidoglycan cell wall, (b) cytoplasmic membrane, where chemical and physical properties are modified and results in an imbalance of osmolality, permeability, electron transport, and cellular breathing and (c) ribosomal DNA, molecular sites of phosphorus and sulfur present in proteins, especially in enzymes involved in the electron transport chain [7].

Currently there is a recommendation by International Organization for Standardization (ISO), a worldwide federation of national standards [8, 9], for evaluating potency of silver nanoparticles against cell wall degradation of *Staphylococcus aureus* and muramic acid release, using gas chromatography-mass spectrometry (GC-MS) [8, 10].

However, studies in the literature have used the methods recommended for antimicrobial drugs: diffusion in agar and determination of minimum inhibitory concentration (MIC) [11–13] and minimum bactericidal concentration (MBC) according to the current Clinical and Laboratory Standards Institute (CLSI) document recommendation [14].

The aim of this study was to evaluate the in vitro activity of AgNPs stabilized with different compounds against MDR and antimicrobial-susceptible Gram-positive and Gram-negative microorganisms, using different methods.

## Results and discussion

### AgNPs

**Fig. 1:**
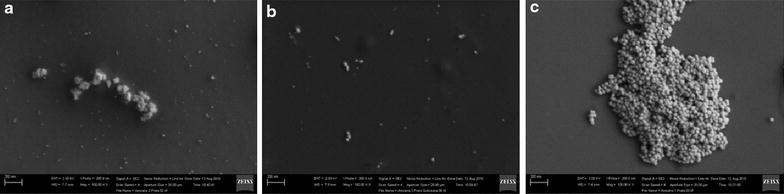
FEG-SEM micrographs of AgNPs stabilized by **a** PVA, **b** chitosan and **c** citrate

**Fig. 2:**
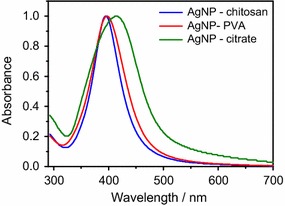
UV-vis spectroscopy spectra of PVA, chitosan and citrate AgNPs

The size and morphology of the AgNPs were investigated by Field Emission Gun Scanning Electron Microscope–FEG-SEM (Fig. 1). PVA-AgNPs and Chitosan-AgNPs presented an average diameter around 10 and 25 nm, respectively, while the citrate-AgNPs have around 40 nm of diameter. The UV–VIS spectra in Fig. 2 show the optical properties of PVA, Chitosan and Citrate stabilized AgNPs. The results reveal the surface plasmon resonances peaks around 400 nm for all systems, which is typical for nanostructured silver [15–17]. It is well known that the surface plasmon resonances depend strongly on the size, shape and functionalization of the metallic NPs [18]. The increasing in the size of the AgNPs leads to a red-shift and broadening of the plasmon resonance band [18], which agree with the spectrum observed for Citrate-AgNPs. The zeta potential analysis reveals significant differences in the surface charges between the three systems (Table 1). Chitosan AgNPs presented high positively surface charge (+41.1 mV), while the citrate ones were very negative (−48.4 mV) and PVA AgNPs were more close to zero (−17.0 mV).

**Table 1:** Zeta potential for PVA, chitosan and citrate AgNPs

AgNPs	Potential zeta
AgNP–PVA	−17.0 mV
AgNP–Chitosan	+41.1 mV
AgNP–Citrate	−48.4 mV

**Fig. 3:**
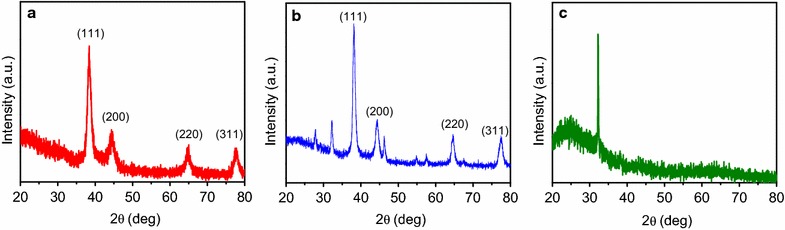
X-ray diffraction patterns for AgNPs stabilized by **a** PVA, **b** chitosan and **c** citrate

Figure 3 shows the x-ray diffraction spectra of the AgNPs stabilized by PVA, chitosan and citrate. The spectra of PVA AgNP and chitosan AgNP reveals the existence of the peaks at 2θ = 38.15°, 44.34°, 64.5° and 77.46°, which can be assigned to the (111), (200), (220), and (311) reflections of the face centered cubic (fcc) structure of metallic silver, respectively [19–21]. No impurities were detected in the XRD profile of AgNPs-PVA. However, some intense peaks at 2θ angles of 32.2°, 46.3°, 54.7° and 57.3° were observed in the XRD profile of AgNPs-chitosan. Several studies attribute the presence of these small additional peaks to the crystalline organic phase [22, 23]. On the other side, the peaks at 32.2° and 54.7° also match with the diffraction profile of the Ag_2_O [24, 25] and might be an indicate that some silver oxide have been formed during the synthesis or the drying process of AgNPs in presence of chitosan.

The spectrum of citrate AgNPs shows no peak associate to metallic nanoparticle. The peak around 32° can be can be assigned to silver salt. Since we observed the formation of the silver nanoparticles in all our previous characterizations such as FEG-SEM and UV–VIS spectroscopy, this finding suggests that the metallic silver were oxidized to ions during the drying process and implies that the sodium citrate is not a good stabilizer to protect the silver particles against oxidation.

The diameter of the particles (d) stabilized with PVA and chitosan were estimated by Scherrer equation (Eq. 1), where 0.94 is the constant value used for spherical particle shape, λ is the X-ray wavelength (0.15406 nm), B is the line broadening at half maximum intensity of the selected reflection plane in radians, and θ is the Bragg angle.

1 $$d = \frac{0.94 \lambda }{B \cos \theta }$$

The mean crystalline particles size were determined from the main (111) diffraction peak (2θ = 38.15°) of the X-ray diffraction pattern. The diameter found for PVA was 7.1 nm, which is close to the diameter found by SEM images. However, the calculated diameter for chitosan-AgNPs were around 11.9 nm, while the one obtained from the SEM images were 25 nm. This difference might be related to the oxidation of silver ions during the drying process, which was showed in the X-ray pattern, and consequently decrease in the nanoparticle size.

### Growth inhibition by diffusion

**Fig. 4:**
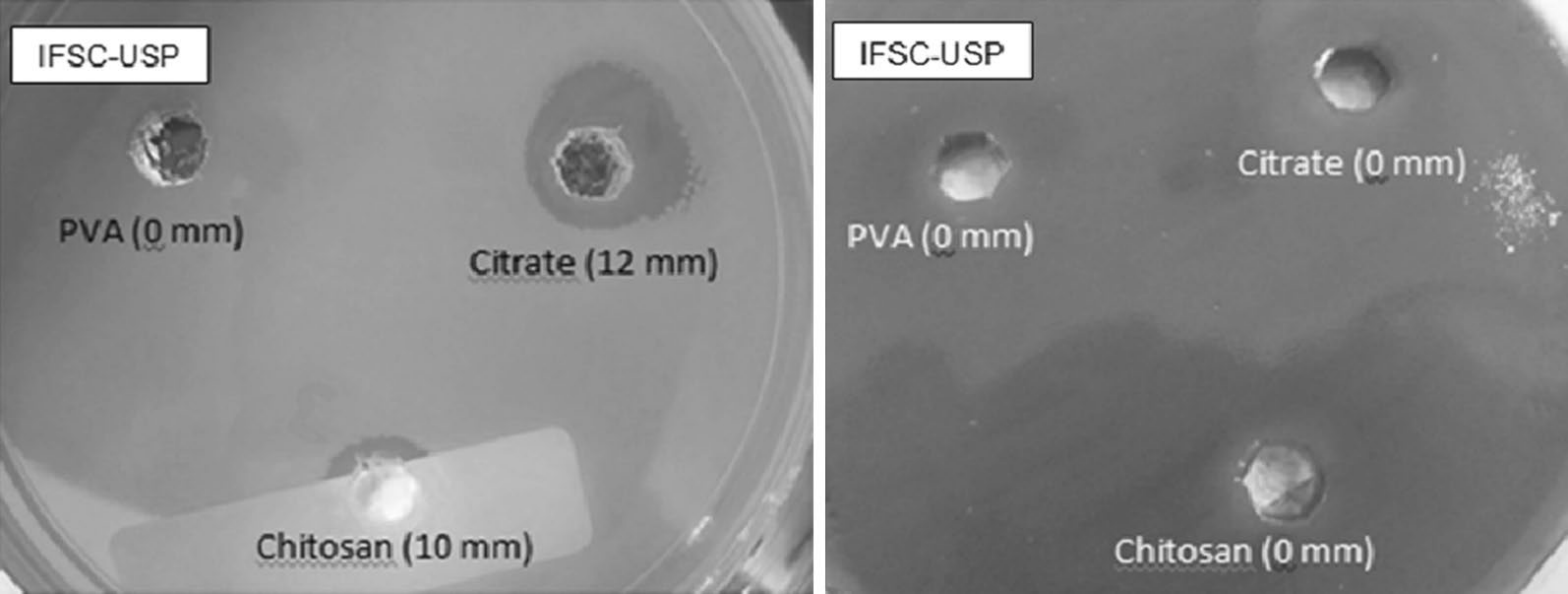
Inhibition by diffusion in depth with AgNPs particles synthesized by IFSC-USP (citrate, chitosan and PVA AgNPs), for *P. aeruginosa* INCQS 230 in agar MHA (*left*) and the same microorganism in MHA blood 5 % (*right*)

**Fig. 5:**
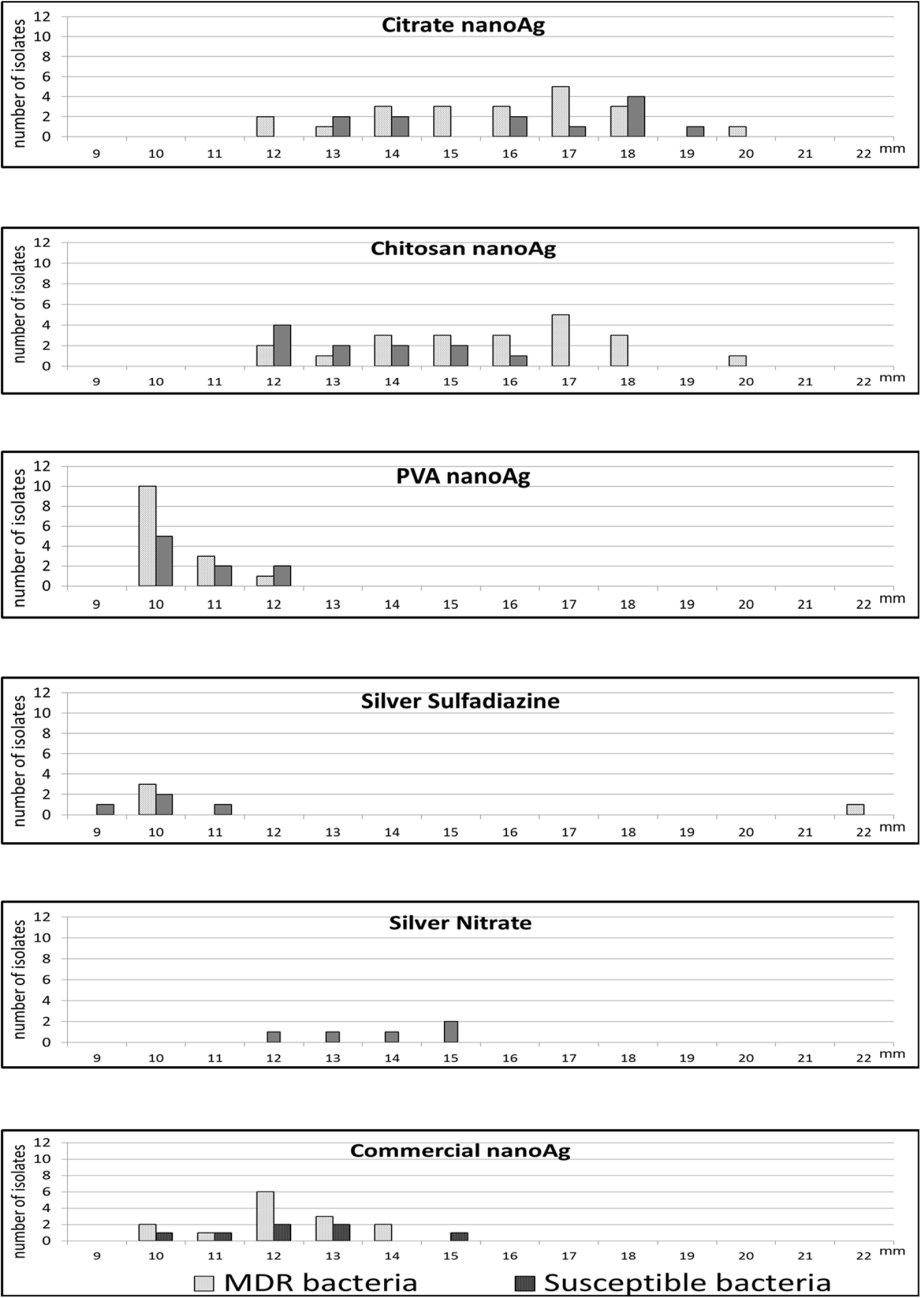
Distribution of size of inhibition zones (mm) obtained by diffusion in depth with AgNPs (citrate, chitosan and PVA) and controls against multidrug-resistant (MDR) (n = 54) and antimicrobial-susceptible bacteria (n = 36). *Asterisk* Only inhibition zones >6 mm were presented. All silver compounds showed absence of inhibition zones (<6 mm) when tested in MHA 5 % blood against the same microorganisms

All inhibition zones produced were plotted according to their size (Fig. 4). The inhibition zone was similar comparing MDR and susceptible bacteria, with larger zones for citrate and chitosan AgNPs than PVA AgNPs. Silver nitrate had no activity against MDR by this method (Fig. 5).

### Determination of minimum inhibitory concentration (MIC)

**Fig. 6:**
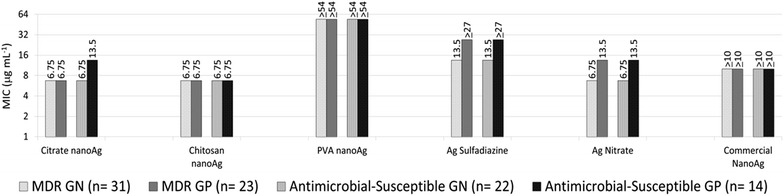
MIC_90_ for AgNPs (citrate, chitosan and PVA) and controls comparing the subgroups MDR Gram-negative GN (n = 31) versus MDR Gram-positive GP (n = 23) (totalizing n = 54) and antimicrobial-susceptible GN (n = 22) versus GP (n = 14) (totalizing n = 36)

The analysis of MIC_90_ results were grouped in MDR and susceptible isolates (Table 2), the results against Gram-positive and Gram-negative were similar for chitosan AgNPs (6.75 μg mL^−1^), PVA AgNPs (≥54 μg mL^−1^) and commercial AgNPs (≥10 μg mL^−1^). The MIC_90_ for citrate AgNPs was higher against susceptible Gram-positive isolates (13.5 μg mL^−1^) than against susceptible Gram-negative and MDR (6.75 μg mL^−1^). Regarding the silver controls, Ag sulfadiazine and Ag nitrate, the MIC_90_ were respectively ≥27 and 13.5 μg mL^−1^, for Gram-positive isolates and 13.5 and 6.75 μg mL^−1^ for Gram-negative, independent of its antimicrobial resistance profile. In all tests the control silver sulfadiazine showed higher MIC_90_ results than citrate and chitosan AgNPs, while silver nitrate showed similar results to citrate AgNPs, except for a lower inhibitory effect against MDR Gram-positive (13.5 μg mL^−1^). It was not possible to define the exact MIC_90_ values for commercial AgNPs (≥10 μg mL^−1^) and PVA AgNPs, the less potent AgNPs particle evaluated was ≥54 μg mL^−1^ (Fig. 6). Summarizing, the effect of AgNPs was similar regardless of antimicrobial resistance profile and showed better activity against Gram-negative.

**Table 2:** MIC, MIC_50_, MIC_90_ and MBC for MDR (n = 56) and antimicrobial susceptible microorganisms (n = 34)

Microorganism	Citrate		Chitosan		PVA		Ag Sulfadiazin		Ag Nitrate		Commercial AgNPs	
	MIC	MBC	MIC	MBC	MIC	MBC	MIC	MBC	MIC	MBC	MIC	MBC
*A. baumannii* MDR (A1)	3.4	13.5	6.7	6.7	≥54	≥54	6.7	6.7	3.4	3.4	≥10	≥10
*A. baumannii* MDR (A2)	3.4	3.4	6.7	6.7	27	27	6.7	6.7	3.4	3.4	≥10	≥10
*A. baumannii* MDR (A3)	3.4	13.5	6.7	6.7	13.5	13.5	6.7	6.7	3.4	3.4	≥10	≥10
*A. baumannii* MDR (A4)	3.4	13.5	6.7	6.7	≥54	≥54	13.5	13.5	3.4	3.4	≥10	≥10
*A. baumannii* MDR (A5)	3.4	13.5	6.7	6.7	13.5	13.5	6.7	6.7	3.4	3.4	≥10	≥10
*A. baumannii* MDR (A6)	3.4	13.5	6.7	6.7	13.5	13.5	6.7	6.7	3.4	3.4	≥10	≥10
*A. baumannii* MDR (A7)	3.4	27	6.7	6.7	13.5	27	6.7	13.5	3.4	13.5	≥10	≥10
*A. baumannii* MDR (A8)	3.4	27	6.7	27	13.5	13.5	6.7	13.5	3.4	13.5	≥10	≥10
*A. baumannii* MDR (A9)	3.4	27	6.7	6.7	13.5	13.5	6.7	6.7	3.4	3.4	≥10	≥10
*A. baumannii* MDR (A10)	3.4	3.4	6.7	6.7	13.5	13.5	13.5	13.5	3.4	3.4	≥10	≥10
*A. baumannii* MDR (A11)	3.4	13.5	6.7	6.7	13.5	27	6.7	6.7	3.4	3.4	≥10	≥10
*A. baumannii* MDR (A12)	3.4	3.4	6.7	6.7	27	27	13.5	13.5	3.4	3.4	≥10	≥10
MIC_50_ *A. baumannii* MDR	3.4	–	6.7	–	13.5	–	6.7	–	3.4	–	≥10	–
MIC_90_ *A. baumannii* MDR	3.4	–	6.7	–	≥54	–	13.5	–	3.4	–	≥10	–
*A. baumannii* S (AS1)	3.4	3.4	3.4	6.7	6.7	13.5	6.7	6.7	3.4	3.4	≥10	≥10
*A. baumannii* S (AS2)	1.6	3.4	1.6	1.6	6.7	13.5	6.7	13.5	3.4	3.4	≥10	≥10
*A. baumannii* S (AS3)	3.4	3.4	3.4	3.4	6.7	13.5	6.7	6.7	3.4	3.4	≥10	≥10
*A. baumannii* S (AS4)	3.4	3.4	3.4	3.4	27	27	6.7	6.7	3.4	3.4	≥10	≥10
*A. baumannii* S (AS5)	3.4	3.4	3.4	6.7	≥54	≥54	6.7	6.7	3.4	3.4	≥10	≥10
MIC_50_ *A. baumannii* S	3.4	–	3.4	–	6.7	–	6.7	–	3.4	–	≥10	–
MIC_90_ *A. baumannii* S	3.4	–	3.4	–	≥54	–	6.7	–	3.4	–	≥10	–
*S. maltophilia* MDR (MO9)	1.6	13.5	6.7	6.7	≥54	≥54	6.7	6.7	3.4	3.4	≥10	≥10
*S. maltophilia* MDR (MO11)	1.6	3.4	6.7	6.7	≥54	≥54	6.7	6.7	6.7	6.7	≥10	≥10
MIC_50_ *S. maltophilia* MDR	1.6	–	6.7	–	≥54	–	6.7	–	3.4	–	≥10	–
MIC_90_ *S. maltophilia* MDR	1.6	–	6.7	–	≥54	–	6.7	–	6.7	–	≥10	–
*P. aeruginosa* MDR (P1)	3.4	3.4	6.7	13.5	27	≥54	6.7	6.7	1.6	1.6	≥10	≥10
*P. aeruginosa* MDR (P2)	3.4	6.7	6.7	13.5	13.5	27	13.5	13.5	3.4	6.7	≥10	≥10
*P. aeruginosa* MDR (P3)	3.4	3.4	6.7	13.5	13.5	27	13.5	13.5	3.4	3.4	≥10	≥10
*P. aeruginosa* MDR (P4)	3.4	13.5	6.7	13.5	13.5	≥54	13.5	13.5	6.7	≥27	≥10	≥10
*P. aeruginosa* MDR (P5)	3.4	3.4	6.7	13.5	13.5	27	6.7	6.7	3.4	6.7	≥10	≥10
MIC_50_ *P. aeruginosa* MDR	3.4	–	6.7	–	13.5	–	13.5	–	3.4	–	≥10	–
MIC_90_ *P. aeruginosa* MDR	3.4	–	6.7	–	27	–	13.5	–	6.7	–	≥10	–
*P. aeruginosa* S (PS1)	3.4	34	6.7	13.5	13.5	13.5	6.7	6.7	3.4	13.5	≥10	≥10
*P. aeruginosa* S (PS2)	3.4	3.4	6.7	6.7	13.5	≥54	13.5	13.5	6.7	13.5	≥10	≥10
*P. aeruginosa* S (PS3)	3.4	1.6	3.4	3.4	≥54	≥54	13.5	13.5	6.7	6.7	≥10	≥10
*P. aeruginosa* S (PS4)	1.6	1.6	3.4	6.7	13.5	13.5	3.4	13.5	1.6	3.4	≥10	≥10
*P. aeruginosa* S (PS5)	1.6	3.4	6.7	13.5	13.5	27	6.7	6.7	3.4	3.4	≥10	≥10
*P. aeruginosa* S (PS6)	1.6	3.4	6.7	13.5	6.7	≥54	6.7	13.5	3.4	3.4	≥10	≥10
*P. aeruginosa* S (PS7)	1.6	3.4	3.4	13.5	6.7	13.5	13.5	13.5	6.7	6.7	≥10	≥10
*P. aeruginosa* ATCC 27853 S (MO18)	3.4	6.7	13.5	13.5	13.5	≥54	6.7	6.7	3.4	3.4	≥10	≥10
MIC_50_ *P. aeruginosa* S	1.6	–	6.7	–	13.5	–	6.7	–	3.4	–	≥10	–
MIC_90_ *P. aeruginosa* S	3.4	–	13.5	–	≥54	–	13.5	–	6.7	–	≥10	–
Enterobacteriaceae MDR (K1)	6.7	6.7	6.7	6.7	≥54	≥54	13.5	13.5	6.7	≥27	≥10	≥10
Enterobacteriaceae MDR (K2)	6.7	6.7	6.7	6.7	≥54	≥54	13.5	13.5	6.7	6.7	≥10	≥10
Enterobacteriaceae MDR (K3)	6.7	13.5	6.7	6.7	13.5	≥54	13.5	13.5	6.7	6.7	≥10	≥10
Enterobacteriaceae MDR (K4)	6.7	6.7	6.7	6.7	≥54	≥54	13.5	13.5	6.7	6.7	≥10	≥10
Enterobacteriaceae MDR (K5)	6.7	6.7	6.7	6.7	27	≥54	13.5	13.5	6.7	6.7	≥10	≥10
Enterobacteriaceae MDR (K6)	6.7	13.5	6.7	6.7	27	≥54	13.5	13.5	6.7	6.7	≥10	≥10
Enterobacteriaceae MDR (K7)	6.7	6.7	6.7	6.7	27	27	13.5	13.5	6.7	6.7	≥10	≥10
Enterobacteriaceae MDR (K9)	6.7	6.7	6.7	6.7	≥54	≥54	13.5	13.5	6.7	6.7	≥10	≥10
Enterobacteriaceae MDR (K14)	6.7	27	6.7	13.5	≥54	≥54	13.5	13.5	6.7	≥27	≥10	≥10
Enterobacteriaceae MDR (MO13)	3.3	3.3	3.3	3.3	13.5	13.5	13.5	13.5	6.7	≥27	≥10	≥10
Enterobacteriaceae MDR (MO8)	3.3	13.5	6.7	6.7	≥54	≥54	13.5	13.5	3.4	3.4	≥10	≥10
Enterobacteriaceae MDR (MO10)	3.3	3.3	6.7	6.7	≥54	≥54	13.5	13.5	6.7	6.7	≥10	≥10
MIC_50_ Enterobacteriaceae MDR	6.7	–	6.7	–	≥54	–	13.5	–	6.7	–	≥10	–
MIC_90_ Enterobacteriaceae MDR	6.7	–	6.7	–	≥54	–	13.5	–	6.7	–	≥10	–
Enterobacteriaceae S (ENB1)	3.4	3.4	6.7	6.7	27	27	13.5	13.5	6.7	6.7	≥10	≥10
Enterobacteriaceae S (ENB2)	6.7	6.7	3.4	6.7	≥54	≥54	13.5	13.5	13.5	13.5	≥10	≥10
Enterobacteriaceae S (ENB3)	3.4	3.4	3.4	3.4	13.5	13.5	13.5	13.5	3.4	6.7	≥10	≥10
Enterobacteriaceae S (ENB4)	6.7	6.7	3.4	6.7	27	27	13.5	13.5	6.7	≥27	≥10	≥10
Enterobacteriaceae S (ENB5)	6.7	6.7	6.7	6.7	27	27	13.5	≥27	6.7	6.7	≥10	≥10
Enterobacteriaceae S (ENB6)	6.7	6.7	6.7	6.7	27	27	13.5	13.5	6.7	6.7	≥10	≥10
Enterobacteriaceae S (ENB7)	6.7	6.7	3.4	3.4	13.5	13.5	13.5	13.5	6.7	6.7	≥10	≥10
Enterobacteriaceae S (ENB8)	3.4	3.4	3.4	3.4	13.5	27	13.5	13.5	6.7	6.7	≥10	≥10
Enterobacteriaceae S (ENB9)	3.4	27	3.4	3.4	≥54	≥54	13.5	13.5	6.7	6.7	≥10	≥10
MIC_50_ Enterobacteriaceae S	6.7	–	3.4	–	27	–	13.5	–	6.7	–	≥10	–
MIC_90_ Enterobacteriaceae S	6.7	–	6.7	–	≥54	–	13.5	–	13.5	–	≥10	–
*S. aureus* MRSA (S1)	6.7	6.7	3.4	27	≥54	≥54	13.5	13.5	13.5	≥27	≥10	≥10
*S. aureus* MRSA (S2)	6.7	13.5	3.4	6.7	≥54	≥54	≥27	≥27	13.5	≥27	≥10	≥10
*S. aureus* MRSA (S3)	6.7	13.5	3.4	3.4	≥54	≥54	≥27	≥27	13.5	≥27	≥10	≥10
*S. aureus* MRSA (S4)	6.7	13.5	3.4	3.4	≥54	≥54	≥27	≥27	13.5	≥27	≥10	≥10
*S. aureus* MRSA (S5)	6.7	13.5	3.4	3.4	≥54	≥54	13.5	13.5	13.5	13.5	≥10	≥10
*S. aureus* MRSA (S6)	6.7	13.5	3.4	27	≥54	≥54	≥27	≥27	13.5	13.5	≥10	≥10
*S. aureus* MRSA (S7)	6.7	13.5	3.4	3.4	≥54	≥54	≥27	≥27	13.5	13.5	≥10	≥10
*S. aureus* MRSA (S8)	6.7	13.5	3.4	3.4	27	27	≥27	≥27	13.5	13.5	≥10	≥10
*S. aureus* MRSA (MO1)	6.7	27	3.4	27	≥54	≥54	≥27	≥27	13.5	13.5	≥10	≥10
*S. aureus* MRSA (MO2)	6.7	13.5	3.4	3.4	≥54	≥54	13.5	≥27	13.5	13.5	≥10	≥10
*S. aureus* MRSA (MO3)	6.7	13.5	6.7	6.7	≥54	≥54	≥27	≥27	13.5	13.5	≥10	≥10
*S. aureus* MRSA (MO4)	6.7	13.5	6.7	27	≥54	≥54	≥27	≥27	13.5	≥27	≥10	≥10
*S. aureus* MRSA (MO5)	6.7	13.5	6.7	27	≥54	≥54	≥27	≥27	13.5	13.5	≥10	≥10
*S. aureus* MRSA (MO6)	13.5	27	6.7	6.7	≥54	≥54	≥27	≥27	13.5	13.5	≥10	≥10
*S. aureus* MRSA (MO7)	13.5	13.5	6.7	6.7	≥54	≥54	≥27	≥27	13.5	13.5	≥10	≥10
MIC_50_ *S. aureus* MRSA	6.7	–	3.4	–	≥54	–	≥27	–	13.5	–	≥10	–
MIC_90_ *S. aureus* MRSA	13.5	–	6.7	–	≥54	–	≥27	–	13.5	–	≥10	–
*S. aureus* MSSA (MSSA1)	13.5	27	6.7	27	≥54	≥54	≥27	≥27	13.5	13.5	≥10	≥10
*S. aureus* MSSA (MSSA2)	6.7	13.5	6.7	6.7	≥54	≥54	≥27	≥27	13.5	13.5	≥10	≥10
*S. aureus* MSSA (MSSA3)	6.7	13.5	6.7	6.7	≥54	≥54	≥27	≥27	13.5	13.5	≥10	≥10
*S. aureus* MSSA (MSSA4)	6.7	13.5	6.7	≥54	≥54	≥54	≥27	≥27	13.5	13.5	≥10	≥10
*S. aureus* MSSA (MSSA5)	6.7	13.5	6.7	6.7	≥54	≥54	≥27	≥27	13.5	13.5	≥10	≥10
*S. aureus* MSSA (MSSA6)	6.7	13.5	6.7	6.7	≥54	≥54	≥27	≥27	13.5	13.5	≥10	≥10
*S. aureus* ATCC29213 (MO17)	13.5	27	3.4	6.7	≥54	≥54	≥27	≥27	13.5	≥27	≥10	≥10
*S. epidermidis* INCQS198 (MO16)	6.7	6.7	6.7	6.7	27	27	6.7	13.5	6.7	6.7	≥10	≥10
MIC_50_ *S. aureus* MSSA	6.7	–	6.7	–	≥54	–	≥27	–	13.5	–	≥10	–
MIC_90_ *S. aureus* MSSA	13.5	–	6.7	–	≥54	–	≥27	–	13.5	–	≥10	–
*Enterococcus* sp. VRE (V1)	6.7	27	6.7	27	≥54	≥54	≥27	≥27	13.5	13.5	≥10	≥10
*Enterococcus* sp. VRE (V2)	6.7	27	6.7	6.7	≥54	≥54	≥27	≥27	13.5	13.5	≥10	≥10
*Enterococcus* sp. VRE (V3)	6.7	27	6.7	6.7	≥54	≥54	≥27	≥27	13.5	13.5	≥10	≥10
*Enterococcus* sp. VRE (V4)	6.7	27	6.7	13.5	≥54	≥54	≥27	≥27	13.5	13.5	≥10	≥10
*Enterococcus* sp. VRE (V5)	6.7	27	6.7	13.5	27	≥54	≥27	≥27	13.5	13.5	≥10	≥10
*Enterococcus* sp. VRE (V6)	6.7	13.5	3.4	13.5	≥54	≥54	≥27	≥27	6.7	13.5	≥10	≥10
*Enterococcus* sp. VRE (V7)	6.7	27	3.4	6.7	27	≥54	≥27	≥27	6.7	13.5	≥10	≥10
*Enterococcus* sp. VRE (V8)	6.7	27	6.7	6.7	13.5	13.5	≥27	≥27	13.5	13.5	≥10	≥10
MIC_50_ *Enterococcus* sp. VRE	6.7	–	6.7	–	≥54	–	≥27	–	13.5	–	≥10	–
MIC_90_ *Enterococcus* sp. VRE	6.7	–	6.7	–	≥54	–	≥27	–	13.5	–	≥10	–
*Enterococcus* sp. VSE (VSE1)	6.7	27	6.7	13.5	27	≥54	≥27	≥27	≥27	≥27	≥10	≥10
*Enterococcus* sp. VSE (VSE2)	6.7	6.7	6.7	13.5	≥54	≥54	13.5	≥27	≥27	≥27	≥10	≥10
*Enterococcus* sp. VSE (VSE3)	13.5	13.5	3.4	3.4	≥54	≥54	≥27	≥27	≥27	≥27	≥10	≥10
*Enterococcus* sp. VSE (VSE4)	6.7	6.7	6.7	6.7	≥54	≥54	13.5	13.5	13.5	13.5	≥10	≥10
*Enterococcus* sp. VSE (VSE5)	6.7	6.7	6.7	6.7	≥54	≥54	13.5	13.5	13.5	13.5	≥10	≥10
*Enterococcus* sp. VSE (VSE6)	6.7	6.7	6.7	6.7	≥54	≥54	13.5	≥27	13.5	≥27	≥10	≥10
MIC_50_ *Enterococcus* sp. VSE	6.7	–	6.7	–	≥54	–	13.5	–	13.5	–	≥10	–
MIC_90_ *Enterococcus* sp. VSE	13.5	–	6.7	–	≥54	–	≥27	–	≥27	–	≥10	–
MDR (N = 54)												
MIC_50_ MDR	6.7	–	6.7	–	≥54	–	13.5	–	6.7	–	≥10	–
MIC_90_ MDR	6.7	–	6.7	–	≥54	–	≥27	–	13.5	–	≥10	–
Susceptible (N = 36)												
MIC_50_ Susceptible	6.7	–	6.7	–	≥54	–	13.5	–	6.7	–	≥10	–
MIC_90_ Susceptible	6.7	–	6.7	–	≥54	–	≥27	–	13.5	–	≥10	–
TOTAL (N = 90)												
MIC_50_	6.7	–	6.7	–	≥54	–	13.5	–	6.7	–	≥10	–
MIC_90_	6.7	–	6.7	–	≥54	–	≥27	–	13.5	–	≥10	–

### Determination of minimum bactericidal concentration (MBC)

**Fig. 7:**
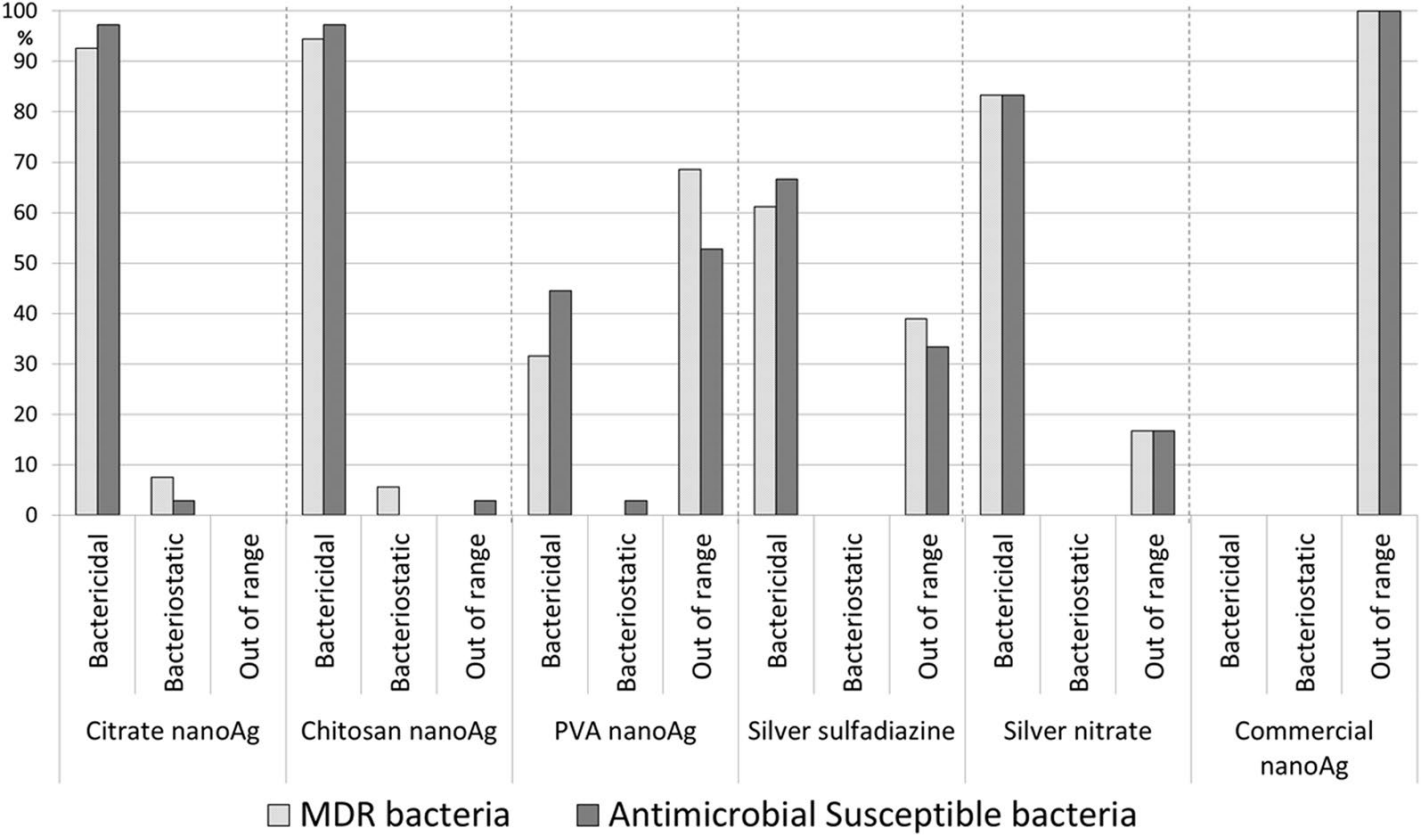
Results of minimum bactericidal concentration/minimum inhibitory concentration (MBC/MIC) ratio of AgNPs particles (citrate, chitosan and PVA) and controls against multidrug-resistant (MDR) (n = 54) and susceptible (n = 36) bacteria

Citrate AgNPs and chitosan AgNPs had higher bactericidal effect than PVA AgNPs and all controls. Both citrate and chitosan achieved bactericidal effect over 97 % of antimicrobial-susceptible bacteria and 93 and 94 % respectively for MDR. For controls, silver nitrate was bactericidal over 83 % of both MDR and antimicrobial-susceptible bacteria while silver sulfadiazine was 61 and 67 % respectively (Fig. 7). Citrate AgNPs were bacteriostatic against three *A. baumannii* MDR isolates, with MBC/MIC ≥8 [26]. The results showed higher bactericidal effect against antimicrobial-susceptible bacteria than against MDR (Table 2).

#### Time-kill

**Fig. 8:**
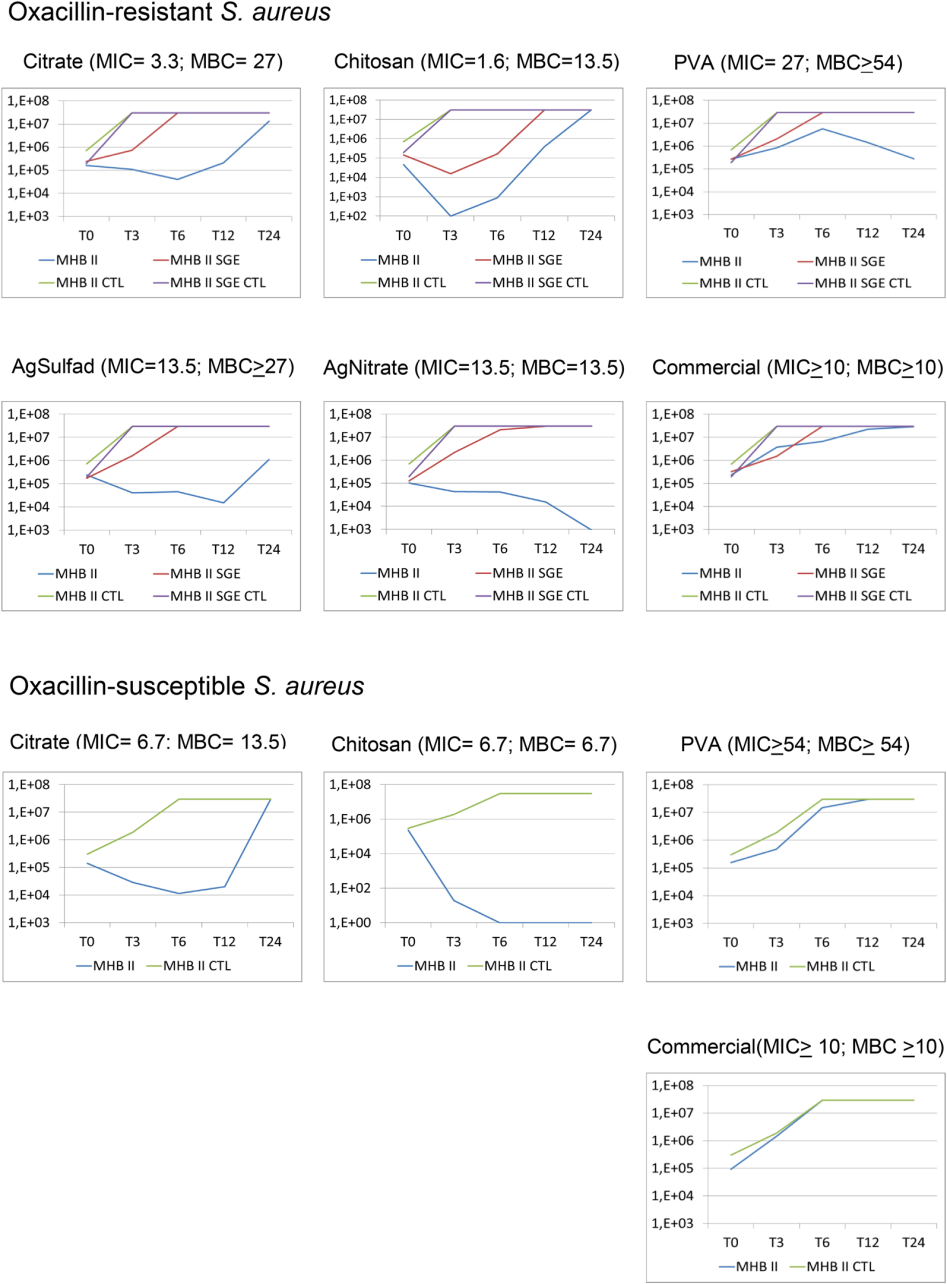
Comparison of time-kill curves for one oxacillin-resistant *S. aureus* (MRSA) and one oxacillin-suscpetible *S. aureus* (MSSA) isolate, using AgNPs particles (citrate, chitosan and PVA) and controls (silver sulfadiazine, silver nitrate and commercial AgNPs). For MRSA was made the comparison using MHB II broth and MHB II blood 1.25 %. MHB II- Mueller–Hinton Broth cation adjusted and microorganism; MHB II CTL-control without microorganisms. MHB II SGE-microorganism and broth enriched with blood; MHB II CTL SGE-only broth and blood. For oxacilin-susceptible *S. aureus* (MSSA3), silver sulfadiazine and silver nitrate curves were not done due to high MICs (Ag Sulfad and Ag Nitrate: MIC ≥27 μg mL^−1^; MBC ≥27 μg mL^−1^)

**Fig. 9:**
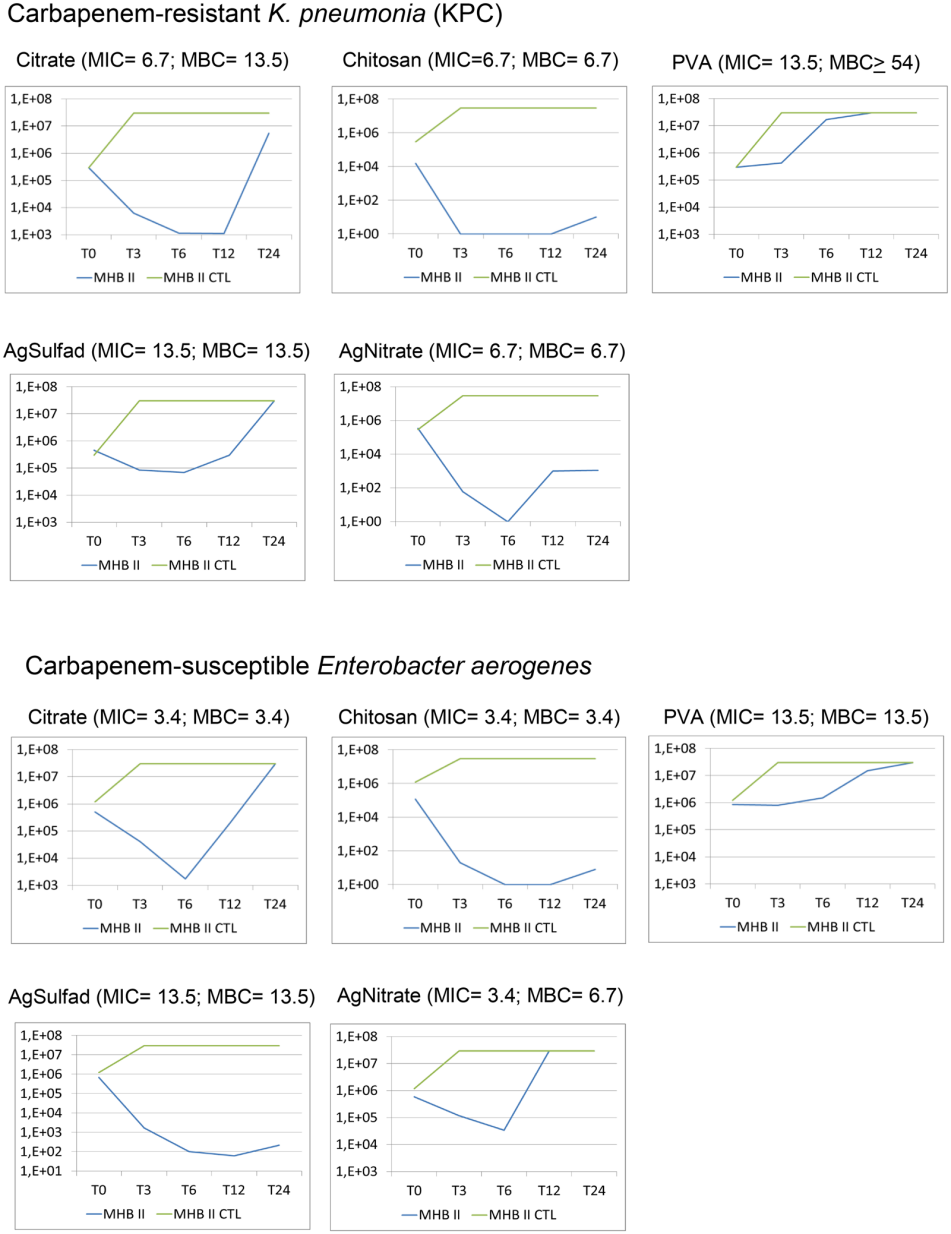
Comparison of time-kill curves for a carbapenem-resistant *K. pneumoniae* (KPC) isolate and an isolate of carbapenem-susceptible *E. aerogenes*, using AgNPs particles (citrate, chitosan and PVA) and controls (silver sulfadiazine and silver nitrate). MHB II- Mueller Hinton Broth cation adjusted and microorganism; MHB II CTL- control without microorganisms. For commercial AgNPs control the curve was not done due to high MICs (MIC ≥ 10 μg mL^−1^; MBC ≥ 10 μg mL^−1^)

The most significant reduction in the number of cfu in the shortest period of time occurred using chitosan AgNPs against the MSSA isolate (4 dilutions) and MRSA isolate (3 dilutions). After 12 h the MDR microorganism decreased its multiplication reaching the initial number of cfu, while the multiplication of antimicrobial-susceptible isolates remained until the end of 24 h, the low counts was reached after the 6th hour (Fig. 8). Among Gram-negative the greatest reduction was observed for chitosan AgNPs against MDR-*K. pneumoniae* (4 dilutions) and against the antimicrobial-susceptible isolate of *E. aerogenes* (4 dilutions). Both remained at 10^1^ cfu mL^−1^ at the end of 24 h (Fig. 9).

As expected, the growth of the microorganisms tested in sub-inhibitory concentrations showed similar behavior when compared to positive controls, without AgNPs. The curves also showed the inhibitory effect of blood on the activity of all silver compounds and controls.

The present study evaluated the in vitro activity of three AgNPs stabilized with different compounds, against clinical isolates including MDR bacteria. The activity of AgNPs using diffusion in solid media and the MIC methods showed similar effect against MDR and antimicrobial-susceptible isolates, with a higher effect against Gram-negative isolates. The best inhibitory effect was achieved with citrate and chitosan AgNPs against Gram-negative bacteria.

The AgNPs showed a good in vitro activity against Gram-negative with different mechanism of resistance, including Enterobacteria harboring carbapenemase (KPC), *Acinetobacter* harboring oxacilinases, and *P. aeruginosa* harboring metallo-beta-lactamases. Interesting points of this study were the inclusion of MDR clinical isolates with previously studied mechanisms of resistance, paired with susceptible isolates, and the large number of isolates evaluated.

In an attempt to reduce the selection of resistant microorganisms, antimicrobials should have bactericidal effect which can be determined by the ratio MBC/MIC ≤4 [26]. The present study showed the predominance of the bactericidal effect of AgNPs particles studied, similar to previously described [3]. The best bactericidal effect was achieved with citrate and chitosan AgNPs against susceptible bacteria. Citrate AgNPs showed the highest inhibitory effect against all isolates, but even better over Gram-negative. Chitosan AgNPs showed identical effect against Gram-positive and Gram-negative, independently of its mechanism of resistance. In agreement with the MIC results, the PVA AgNPs had the time kill curve with less inhibition for all isolates evaluated. The most significant reduction in the number of cfu in the shortest period of time was achieved with chitosan AgNPs against MRSA and *K. pneumoniae* harboring KPC.

The ISO Technical Committee on Nanotechnologies (TC 229) has recently published two new standards methods for analysis of antimicrobial effect [8] and toxicity of AgNPs [9]. However, a variety of other methodologies has been used in literature, as described by Jena et al. using colony-forming unit assay [27], Shrivastava et al. used methods of agar dilution [28] and Patil et al. used qualitative method of agar well diffusion to evaluate MIC of AgNPs [29]. This wide variety of methods makes the comparison of results a difficult task.

In the present study, diffusion in solid media, MIC, MBC, and time-kill methods were evaluated using culture media with and without blood. In all tests some degree of interference of the blood on enriched media was detected, with a reduction of all silver (AgNPs, silver sulfadiazine, and silver nitrate) activity against the microorganisms tested. In the presence of blood, we observed smaller inhibition zones, higher MIC and MBC, and a reduction on the inhibitory effect of death curve with reduction. Thus, media supplement with blood should be avoided to evaluate in vitro activity of AgNPs.

The diffusion method in depth by AWD was previously used as screening test to detect in vitro activity of AgNPs against bacteria [11, 13]. It is an easy method to be use in routine microbiology laboratory. The inhibition zones obtained in the present study showed similar AgNPs activity against MDR and susceptible isolates and that citrate and chitosan AgNPs presented greater effect against the isolates similar with the results using micro dilution method. The MIC_90_ results reinforced and validated the diffusion method results. This results can be explained by the fact that AgNPs interaction with three vital components of cells: peptidoglycan cell wall, cytoplasmic membrane and biomolecules like ribosomal DNA and phosphorus and sulfur groups present in proteins [7, 30].

AgNP shows greater capacity and higher surface area-to-volume ratio compared to silver salts [5, 30–32]. It is an aggregate of silver atoms up to 100 nm in diameter and its features change compared to both the ion and the bulk material in their chemical, mechanical, electrical, optical properties, catalytic activity, conductivity and biological effect. Silver is the more effective antimicrobial agent against bacteria, viruses and other eukaryotic microorganisms [6, 33] than other metals such as copper, titanium, magnesium, zinc or gold.

The impact of the nanosize on silver activity against bacteria was already well demonstrated, Morones et al. showed by a mapping analysis using the X-ray energy dispersive spectrometer (EDS) the effect of AgNPs and pure ionic silver against *E. coli* isolates [4]. The silver AgNPs were well distributed through the isolate electrostatically adhered over the cellular membrane and inside the cytoplasm with consequent high inhibition. On the other hand, silver nitrate was noticeably less detectable over the isolate. Our results showed that AgNPs with PVA, a synthetic bio-friendly water-soluble polymer, had the lowest activity in all tests. Probably because of the higher stability of the system PVA AgNPs as demonstrated by XRD (Fig. 3) and/or interaction with the –OH groups of PVA [34].

No impurities were detected in the XRD profile for AgNPs-PVA. However, some others peaks at 2θ angles of 32.2°, 46.3°, 54.7° and 57.3° were observed in the XRD profile for AgNPs-chitosan, which have been attributed in literature to both crystalline organic phase and Ag_2_O residues. Moreover, the spectrum of AgNPs-citrate shows no peak associate to metallic silver, which suggests that the metallic silver were oxidized to ions during the drying process. Together, these results provide evidence that the excellent in vitro inhibitory effect of citrate AgNPs and chitosan AgNPs against susceptible and MDR bacteria probably arises from the rapid oxidation process of these particles and release of Ag^+^ ions and/or the high surface charge of the particles.

Several factors can have impact on the effect of AgNPs against microorganisms such as size, shape, stability and concentration of AgNPs [35]. Apparently the charge of AgNPs causes less interference over the effect. At biological pH values, the overall surface of the bacteria is negatively charged due to the dissociation of an excess number of carboxylic and other groups in the membrane [4]. Some studies reported that electrostatic attraction between negatively charged bacterial cells and positively charged AgNPs is essential for the activity of AgNPs [36]. However, our results for positive charged chitosan AgNP and negative citrate AgNP were equivalent. Sondi and Salopek-sondi showed that negatively charged AgNPs present excellent antibacterial activity against gram negative *E. coli.* [37]. According to the authors, negative AgNPs somehow interact with the bacterial membrane, causing structural changes and degradation [37]. Furthermore, several studies have demonstrated that the antibacterial activity of chitosan can result in a synergic effect in the Chitosan-AgNPs system [38, 39].

Regardless of the AgNPs charge, the growth inhibition of bacteria is related to the formation of Ag^+^ from the surface of the nanoparticles, attacking the membrane lipids and leading to a breakdown of membrane function [40]. In this way, the antibacterial activities of AgNPs are critically dependent on surface oxidation and optimal particle dispersion [41]. Kim et al. demonstrated that the antioxidant N-acetyl cysteine (NAC) could influence antimicrobial activity induced by a slightly negative charged AgNP (surface zeta potential of −0.33 mV) [42]. The inhibitory effect was abolished by the addition of NAC, while NAC alone did not affect the antimicrobial activity. This can be explained by the fact that the nanosize allowed expansion of the contact surface of Ag^+^ with the microorganisms despite the negative AgNP charge [42].

Studies had demonstrated the in vitro activity of AgNPs against Gram-negative and Gram-positive isolates, however the AgNPs activity against Gram-negative is not fully understood [4, 37]. The AgNPs effect against Gram-negative could differ based on the in vitro method used, and few studies used the gold standard method by muramic acid release and quantification by a gas chromatography-mass spectrometry (GC–MS) [8, 10]. Dawy et al. described better AgNPs inhibitory effect against *S. aureus* than *E. coli* and *P. aeruginosa* using agar diffusion method, arguing that the lipopolysaccharide (LPS) could have a protective effect in Gram-negative bacteria [40]. On the other hand, Shrivastava et al. evaluated by agar dilution and growth curve methods two strains of antimicrobial-susceptible microorganisms: *S. aureus* (ATCC 25923) and *E. coli* (ATCC 25922); and two antimicrobial resistant: *E. coli* and *S. typhus*, and showed better activity of AgNPs against Gram-negative [28]. Sondi and Salopek-Sondi studied one prototype of *E. coli* by agar dilution and growth curve and defended that Gram-negative bacteria are more susceptible to AgNPs because the positive charges of Ag^+^ interact with the LPS of the cell membrane with greater affinity when compared to Gram-positive, resulting in the cell membrane pores [37]. Few studies used MIC and or MBC to evaluate the effect of AgNPs against bacteria [11, 43]. Egger et al. evaluated the effect of silica AgNPs and silver nitrate against a small number of Gram-negative (n = 5) and Gram-positive (n = 4) bacteria [43]. The MIC and MBC of these two groups differed significantly with greater susceptibility against Gram-negative. The authors speculating that this effect can be due to the peculiarities of the cell wall of bacteria, Gram-positive contain multiple layers of peptidoglycan (around 30 nm) compared with the wall of Gram-negative bacteria (around 3 nm). Another possibility is the high content of teichoic and lipoteichoic acids common in Gram-positive that have strong negative charge that may hijack free Ag^+^ ions. Our MIC_50_ results against MDR *P. aeruginosa* were 3.37, 6.75 and 13.5 μg/ml respectively for Citrate AgNPs, chitosan and PVA lower than previously reported. Singh et al. using “green synthesized” AgNPs by *Phyllanthus amarus* described MIC_50_ of 6.25 μg/ml [11] and using *Tinospora cordifoliapara* MIC_50_ of 50 μg/ml against *P. aeruginosa* [44]. Ansari et al. studied the action of AgNPs against *S. aureus* and reported MIC_90_ for MSSA of 25 μg/ml and MRSA of 50 μg/ml [5]. These findings were higher than our results that showed MIC_90_ for MSSA/MRSA 13.5 and 6.75 μg/ml respectively for Citrate and chitosan AgNPs. Thus, this issue is still controversy and more studies are need.

Our results showed the great potential of silver AgNPs for clinical use. However, its toxicity and the concern with the risk to the environment, with the growing accumulation of these substances when disposed should be addressed.

## Conclusions

The synthesized AgNPs, especially citrate and chitosan AgNPs, showed excellent in vitro inhibitory effect against susceptible and MDR bacteria. The AgNPs effect was superior against Gram-negative compared to Gram-positive. The inhibitory effect (MIC) was similar against MDR and susceptible bacteria, whit bactericidal (MBC) effect higher against susceptible isolates. It seems that media supplement with blood should be avoided to evaluate in vitro activity of AgNPs. Diffusion method in depth can be used as screening test, and MBC/MIC and time kill as reference methods.

The bactericidal effect of AgNPs can be translated into important therapeutic and clinical options in the future, especially considering the shortage of new antimicrobials against the emerging antimicrobial resistant microorganisms, in particular against Gram-negative bacteria.

## Methods

The study was performed in the Laboratory of Medical Research 54 (LIM-54) of the Faculty of Medicine of the University of São Paulo (FM-USP) and the Institute of research and development of Laboratório Fleury Medicina e Saúde.

### Microorganisms

Silver compounds were studied against a set of 90 clinical microorganisms, including MDR (n = 54) and susceptible microorganisms (n = 36). The MDR belonged to different clones, previously characterized by pulsed field gel electrophoresis (PFGE), and obtained from laboratory strain bank LIM-54. Susceptible strains from Laboratório Alerta (Universidade Federal de São Paulo, UNIFESP) were also included and The *American Type Culture Collection* (ATCC) and the Instituto Nacional de Controle de Qualidade em Saúde (INCQS)were used as control. The following resistance genes had been previously studied in Gram-negative and Gram-positive bacteria: carbapenem and polymyxin B-resistant *A. baumannii* (harboring *oxa*-_*23*_ and *oxa*-_*143*_ genes) (n = 12), carbapenem and polymyxin B-susceptible *A. baumannii* (n = 5), carbapenem-resistant *P. aeruginosa* (harboring SPM and VIM genes) (n = 5), carbapenem-susceptible *P. aeruginosa* (n = 8), carbapenem-resistant Enterobacteriaceae (harboring extending spectrum beta lactamase ESBL and KPC-2 genes) (n = 12), carbapenem-susceptible Enterobacteriaceae (n = 9) and sulfamethoxazole/trimethoprim- and levofloxacin-resistant *S. maltophilia (*harboring *Sul*-_*1*_*and Sul*-_*2*_genes) (n = 2). Oxacillin-resistant *S. aureus* (methicillin resistant *S. aureus* MRSA-SCCmec I, II, III, IVa, IVb and IVc positives) (n = 5), oxacilin-susceptible *S. aureus* (n = 8), vancomycin-resistant *Enterococcus* spp. (harboring v*anA* gene) (n = 8), vancomycin-susceptible *Enterococcus* spp. (n = 6)

### AgNPs

The AgNPs particles (whit citrate, PVA and chitosan) were produced in the Instituto de Física de São Carlos, Universidade de São Paulo (IFSC-USP). All AgNPs were produced from 1 mM of AgNO_3_ (Sigma-Aldrich, St. Louis, MO, USA), resulting in 108 μg mL^−1^ AgNPs.

The amount of capping agents (citrate, chitosan and PVA) used in each synthesis depended on the stabilizing agent and it was optimized in order to have the best stability. Since citrate, PVA and chitosan presents different molecular weight and functionalization mechanism, the capabilities of them to stabilize the silver nanoparticles are different and the ratios between silver and capping agent had to be optimized in each case.

It is worth emphasizing that the main idea of this study was to evaluate which system could present the best antimicrobial activity in terms of surface charge, size, and stability. Since these capping agents are very studied in synthesis of silver nanoparticles and present significant differences, they were chosen for this comparative study.

### Synthesis of PVA, chitosan and citrate AgNPs

#### PVA AgNPs

PVA AgNPs were synthesized based on modification of previous methods [45, 47, 48]. Briefly, 30 mL of a 4 g L^−1^ solution of polyvinyl alcohol (PVA, Mw 89,000–98,000) (Sigma-Aldrich’s. Louis, MO, USA) were mixture to 30 mL of 1 mM of AgNO_3_ (Sigma-Aldrich) at room temperature and under magnetic stirring. After 10 min, 1 mL of a cold solution of 0.1 mol L^−1^ NaBH_4_ (Sigma-Aldrich, St. Louis, MO, USA) were added. The system has passed from transparent color to yellow, indicating the formation of the nanoparticles [47].

#### Chitosan AgNPs

A similar strategy was used to produce the chitosan AgNPs [49]. Briefly, 30 mL of a 1 g L^−1^ chitosan solution (Medium molecular weight, Sigma-Aldrich, St. Louis, MO, USA) containing 1 % acetic acid were added to 30 mL of a 1 mM solution of AgNO_3_ (Sigma-Aldrich, St. Louis, MO, USA) at room temperature and under magnetic stirring, followed by adding 1 mL of 0.1 mol L^−1^ NaBH_4_ cold solution. The color of the system also changed from transparent to yellow.

#### Citrate AgNPs

Citrate AgNPs were synthesized based on previous methods with modifications [46]. For this, 30 mL of 1 mM AgNO_3_ solution (Sigma-Aldrich) were heated to boiling in a round-bottom flask connected to a condenser. After reaching the boiling point, 13 mL of a sodium citrate (Sigma-Aldrich) solution of 1 % w/v were added under vigorous agitation. After a few minutes, the solution color changed from transparent to yellow and the heating system was switched off.

#### Characterization of AgNPs (PVA, chitosan and citrate)

The optical properties of the AgNPs were characterized by Ultraviolet–Visible spectroscopy (UV–VIS) (HITACHI, U-2900) using a 1 cm quartz cell. The size and morphology of the silver nanoparticles were analyzed by Field Emission Gun Scanning Electron Microscope–FEG-SEM (Zeiss) at 2.0 kV. For this, diluted samples of PVA, chitosan and citrate stabilized AgNPs were deposited in silicon substrates without any further coating or processing and dried at room temperature. The surface charge of the particles was evaluated in suspension by zeta potential measurements (Zetasizer Nano, ZS90). The crystal structure of silver nanoparticles stabilized by PVA, chitosan and citrate were studied with X-ray powder diffraction (XRD) in a Rigaku diffractometer, with CuKa (1.5406 Å) radiation. For this, the particles were synthesized according to the described previously, centrifuged to remove the excess of stabilizers and dried at room temperature.

#### Controls

Silver sulfadiazine (Sigma-Aldrich, St. Louis, MO, USA) and silver nitrate (Sigma-Aldrich, St. Louis, MO, USA) at initial concentration of 54 μg mL^−1^ were used as control.

The commercial Sigma-Aldrich AgNPs (St. Louis, MO, USA) at concentration of 20 μg mL^−1^ with 60 nm sized nanoparticles, stabilized by citrate was used as control.

*Tests* The aim of the selected methods was to study the antibacterial effect of AgNPs produced. Their inhibitory and bacteriostatic/bactericide effect, as well as the dynamic of bacterial killing were evaluated using the following tests:

### Growth inhibition by diffusion

The test was carried out according to the agar well diffusion (AWD) method [12, 13] as a screening of AgNPs inhibitory effect. To evaluate possible interference of enriched culture medium the growth inhibition tests in depth were performed using Mueller–Hinton Agar—MHA (Biomerieux, Marcy L’Etoile, France) and MHA with sheep blood 5 % (Difco, Sparks, MD, USA).

The holes made in agar with 5 mm diameter were aseptically filled with 50 μL of AgNPs and controls. The plates were then incubated at 36 ± 1 °C for 24 h and inhibition zones measured, in millimeters, under reflected light.

### Determination of minimum inhibitory concentration (MIC)

The AgNPs inhibitory power against bacteria was evaluated using MIC. The MIC determination was carried out with Mueller–Hinton Broth -MHB II and Trypticase Soy Broth—TSB, and both showed similar results. The impact of blood was evaluated using MHB II 1.25 % sheep blood. The results were affected by the presence of blood, thus MHBII was chosen to perform all the tests.

The samples were diluted in a serial logarithmic base 2 (log_2_), in MHB cation adjusted (MHB II) (BBL, Sparks, MD, USA), plus microbial suspensions with 10^4^ cfu in MHB II (Difco), resulting in a micro dilution panel from 1:4 to 1:256, following the methodology of CLSI document M07-A9 [14]. According to original concentration, after initial 1:4 dilution PVA, chitosan and citrate AgNPs started from 27 μg mL^−1^, the controls silver nitrate and silver sulfadiazine started from 13.5 μgmL^−1^and commercial AgNPs from 5 μg mL^−1^.The 96 micro well plates were incubated under 35 ± 1 °Cfor 16 to 18 h and read visually with transmitted light observing the presence or absence of turbidity. The first well with no microbial growth was defined as the MIC, expressed in μg mL^−1^. To evaluate the reproducibility of the method, some tests were carried out in triplicate for sampling.

### Minimum bactericidal concentration determination (MBC)

The concentration of AgNPs required to achieve the bactericidal effect was defined using MBC. After reading the MIC, all wells without visible turbidity were plated in MHA and incubated under 35 ± 1 °C for 16–18 h. The plates were read visually with reflected light, observing the presence or absence of macroscopic bacterial growth. The lower dilution without macroscopic bacterial growth was defined as MBC. According to CLSI method for antimicrobial drugs, described in the document M7-A9 [14].

The MBC/MIC ratio was used to define the mode of activity of silver derivatives: bactericidal when scores are 1, 2 and 4 or bacteriostatic if score >4 [26].

### Time-kill tests

The dynamic of the AgNPs inhibitory effect was carried out using the time-kill curves under AgNPs concentration equal to 1 × MIC [50]. Four representative strains of MDR and antimicrobial susceptible Gram-positive and Gram-negative were evaluated by this method, one strain of *S. aureus* oxacilin-resistant and one oxacilin-susceptible, *K. pneumonia* carbapenem-resistant and *E. aerogenes* carbapenem-susceptible evaluated according with previous described [51, 52]. Shortly, after know the MIC for each AgNPs and microorganism evaluated were made tubes with 10 ml of MHBII and AgNPs at 1xMIC and 10^5^ cfu/mL of each microorganism . For the negative control, AgNPs were substituted for MHBII. All tubes were incubated under 36 ± 1 °C and the number of viable microorganisms quantified on MHA at time 0, 3, 6, 12 and 24 h. The number of viable cfu in each time was plotted on a graph profiling the time-kill curve, compared to the curve of positive and negative controls.

The time-kill was not performed for the isolates with indeterminate MIC, that is, above the highest concentration tested (≥54 μg mL^−1^, ≥27 μg mL^−1^ e ≥ 10 μg mL^−1^). Except for MRSA isolate against the commercial AgNPs and MSSA isolate against PVA and commercial AgNPs. These were deliberately tested to show the shape of the curves in sub-inhibitory conditions.

To evaluate the blood interference observed in agar diffusion and MIC, for the isolated *S. aureus* MRSA (M01) the curve was held in MHBII broth and MHBII 1.25 % sheep blood broth.

## Abbreviations

AgNPs: silver nanoparticles
ATCC: American Type Culture Collection
CLSI: Clinical and Laboratory Standards Institute
DLS: dynamic light scattering
EDS: energy dispersive spectrometer
ESBL: extended spectrum beta lactamase
FM-USP: Faculty of Medicine of the University of São Paulo
GC–MS: gas chromatography-mass spectrometry
INCQS: Instituto Nacional de Controle de Qualidade em Saúde
ISO: International Organization for Standardization
KPC: *Klebsiella pneumoniae* carbapenemase
LIM: Laboratory of Medical Research
LPS: pipopolysaccharide
MBC: minimum bactericidal concentration
MDR: multidrug resistant microorganisms
MHA: mueller–hinton agar
MHB: mueller hinton broth
MIC: minimum inhibitory concentration
MRSA: methicillin resistant *Staphylococcus aureus*
MSSA: methicillin susceptible *S. aureus*
NAC: *n*-acetyl cysteine
PFGE: pulsed field gel electrophoresis
PVA: polyvinyl alcohol
SCC-mec: staphylococcal cassette chromosome mec
SPM: São Paulo metallo-beta-lactamase
TSB: triptcase soy broth
UNIFESP: Universidade Federal de São Paulo
USP: Universidade de São Paulo
VIM: Verona imipenemase
VRE: vancomycin resistant *Enterococcus*
XRD: X-ray diffraction
